# Growth performance, carcass traits, and breast meat quality of slow-growing broiler strains reared in a free-range system: effects of genotype and sex at different slaughter ages

**DOI:** 10.1007/s11250-026-05328-8

**Published:** 2026-09-08

**Authors:** Marcela Batista Lacerda, Jean Kaique Valentim, Rodolpho de Almeida Torres Filho, Cláudio Vieira de Araújo, Raiane Azeredo Silva, Eugênia Viana Romanelli, Cleube Andrade Boari, Alexander Alexandre de Almeida, Sandra Regina Freitas Pinheiro

**Affiliations:** 1 Department of Animal Science, Federal University of the Jequitinhonha and Mucuri Valleys (UFVJM), Diamantina, MG Brazil; 2 Department of Animal Production, Federal Rural University of Rio de Janeiro (UFRRJ), Seropédica, RJ Brazil; 3 Fluminense Federal University (UFF), Niterói, RJ Brazil; 4 Federal University of Mato Grosso (UFMT), Sinop, MT Brazil; 5 Federal University of Grande Dourados (UFGD), Dourados, MS Brazil

**Keywords:** Free-range broilers, Slow-growing strains, Carcass traits, Meat quality, Growth performance

## Abstract

This study evaluated the effects of strain and sex on growth performance, carcass characteristics, and meat quality of slow-growing broilers reared in a semi-intensive free-range system at different slaughter ages. A total of 864 one-day-old chicks from four commercial slow-growing strains (Pescoço Pelado Hubbard, Pescoço Pelado Sasso, Pesadão Hubbard, and Pesadão Sasso) were assigned to a completely randomized 4 × 2 factorial design (strain × sex). Growth performance was evaluated at 70, 77, and 84 d of age, and carcass traits and meat quality parameters were assessed at each slaughter age. Male birds showed greater body weight, higher feed intake, and better feed conversion ratio than females at all evaluated ages (*P* < 0.05). Pescoço Pelado Hubbard and Pesadão Hubbard strains exhibited superior growth performance compared with the Sasso strains (*P* < 0.05). Females showed greater breast yield, whereas males had higher thigh–drumstick yield (*P* < 0.05). A significant strain × sex interaction was observed for carcass yield at 77 d (*P* < 0.05), with Pescoço Pelado Hubbard males showing greater carcass yield than females of the same strain, whereas no sex differences were observed within the other strains. Meat quality traits differed according to strain and sex at specific slaughter ages, particularly in colour parameters, pH, cooking loss, and shear force (*P* < 0.05). A significant strain × sex interaction was observed for cooking loss at 84 d (*P* < 0.05), with Pesadão Sasso females showing greater cooking loss than males of the same strain, whereas no sex differences occurred within the other strains. These interactions demonstrate that the effect of sex on specific carcass and meat quality traits can vary among genotypes at particular slaughter ages. Overall, Hubbard strains demonstrated superior productive performance under semi-intensive free-range conditions, while the genotype-specific sex responses observed for carcass yield and cooking loss highlight the importance of considering both factors when defining slaughter strategies.

## Introduction

The production of free-range broiler chickens has gained increasing attention due to its potential to add value to poultry products while addressing growing concerns regarding animal welfare and sustainability (Morais et al. 2015; Zidane et al. 2023). Consumer demand for meat produced under welfare-friendly systems has expanded in recent years, creating opportunities for alternative poultry production systems that combine ethical production practices with differentiated product quality.

Genetic improvement has played a central role in poultry production by enhancing growth performance, feed efficiency, carcass yield, and meat quality traits to meet market demands for high-value products (Dourado et al. 2009). In free-range systems, birds are allowed to express natural behaviours, which may positively influence physiological responses and product quality, particularly sensory attributes such as flavour and texture that are increasingly valued by consumers (Castellini et al. 2002; Bashir et al. 2023).

Although slow-growing free-range broilers generally exhibit lower growth rates and reduced yields of prime cuts compared with conventional fast-growing strains, their production may offer advantages associated with improved meat quality, texture, and colour, characteristics that are highly valued in niche markets (Moreira et al. 2003; Santos et al. 2005; Mattioli et al. 2025). In addition, these birds may show greater adaptability to less intensive production environments, including increased disease resistance and lower mortality, which may partially offset reduced productive efficiency (Aksoy et al. 2021).

The evaluation of different genotypes under specific production conditions is essential for decision-making in breeding programmes and for improving production efficiency. Such information is also relevant for developing management strategies capable of meeting consumer expectations for high-quality poultry products obtained under welfare-oriented production systems (Neeteson et al. 2020; Naeem et al. 2026). Moreover, environmental and management factors play a critical role in the productive success of free-range broiler systems, as diet composition, housing conditions, and access to outdoor areas may directly affect growth performance, carcass characteristics, and meat quality (Ekerette et al. 2025; Fanatico et al. 2007; Vorel et al. 2026).

Despite the growing interest in alternative poultry production systems, comparative information regarding the performance of commercial slow-growing strains under free-range conditions remains limited, particularly when considering sex-related differences in productive performance, carcass composition, and meat quality at different slaughter ages. Because genotype and sex may influence nutrient utilisation, tissue deposition, and physiological adaptation to production environments, their combined evaluation is necessary to support more efficient production strategies for alternative poultry systems.

We hypothesised that commercial slow-growing strains would differ in productive performance, carcass yield, and meat quality under free-range conditions, and that these responses would be influenced by sex across slaughter ages.

Therefore, this study evaluated the effects of strain and sex on growth performance, carcass yield, and meat quality of slow-growing broiler chickens reared in a semi-intensive free-range system at different slaughter ages.

## Materials and methods

The experiment was conducted at the Poultry Sector of the Department of Animal Science, Federal University of the Jequitinhonha and Mucuri Valleys (UFVJM), Campus JK, Diamantina, Minas Gerais, Brazil. All experimental procedures involving animals were approved by the Institutional Animal Care and Use Committee of UFVJM (protocol no. 12/2020).

**Fig. 1:**
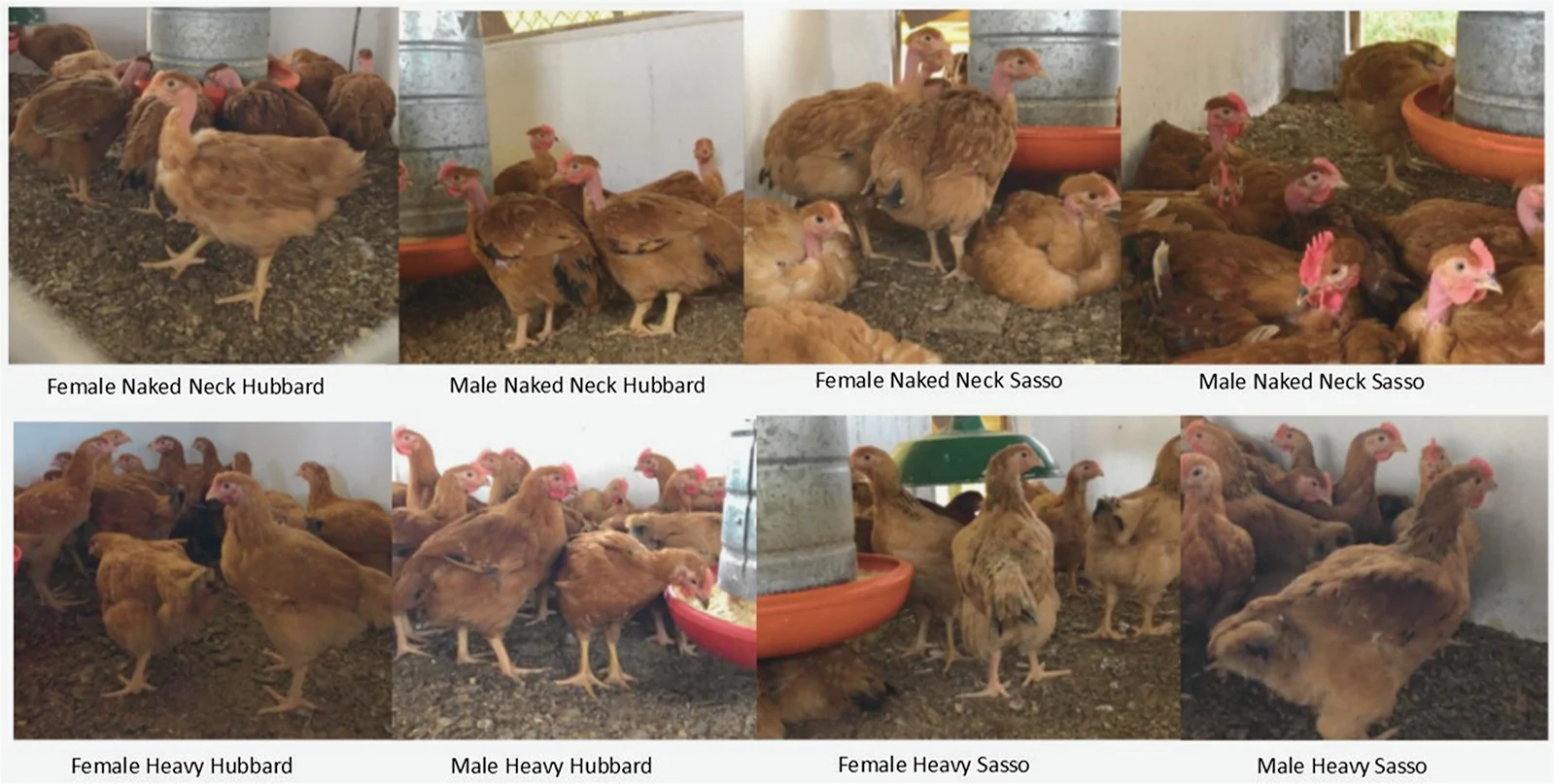
Representative phenotypic characteristics of male and female birds from four slow-growing broiler strains reared in a semi-intensive free-range system

A total of 864 one-day-old slow-growing broiler chicks from four commercial free-range strains (Pesadão Hubbard, Pescoço Pelado Hubbard, Pesadão Sasso, and Pescoço Pelado Sasso) were used (Fig. 1). Birds were distributed in a completely randomised 4 × 2 factorial design (strain × sex), resulting in eight experimental treatments, with four replicates per treatment and 27 birds per experimental unit.

Experimental diets were based on maize and soybean meal and formulated for three feeding phases: starter (1–28 d), grower (29–56 d), and finisher (57–84 d), according to the nutritional recommendations provided by Guabi Nutrição e Saúde Animal S/A for slow-growing free-range broilers (Table 1). Feed and water were supplied ad libitum throughout the experimental period.

**Table 1:** Composition nutrient content of experimental diets fed to slow-growing broilers during the starter, grower, and finisher phases

Nutritional data	Initial	Growth	Final
Metabolizable energy (Kcal/kg)	3,100	3,250	3,300
Calcium (%)	0.950	0.850	0.800
Available phosphorus (%)	0.500	0.470	0.400
Sodium (%)	0.200	0.180	0.180
Crude protein (%)	21.50	19.50	18.50
Digestible arginine (%)	1.342	1.188	1.128
Digestible lysine (%)	1.240	1.180	1.000
Methionine + digestible cystine (%)	0.880	0.790	0.706
Digestible threonine (%)	0.780	0.705	0.630
Digestible tryptophan (%)	0.237	0.209	0.197
Digestible valine (%)	0.903	0.813	0.779

Vitamin–mineral premix supplied per kg of diet: vitamin A, 12,000 IU; vitamin D3, 4,000 IU; vitamin E, 40 IU; vitamin K3, 4 mg; vitamin B1, 11.7 mg; vitamin B2, 6.5 mg; vitamin B6, 3 mg; vitamin B12, 15 µg; folic acid, 1.5 mg; biotin, 0.08 mg; niacin, 50 mg; calcium pantothenate, 14 mg; copper, 3 mg; iron, 15 mg; iodine, 0.20 mg; manganese, 25 mg; selenium, 0.49 mg; zinc, 20 mg

Birds were housed in an experimental masonry poultry house with fibre-cement roofing, divided into 32 floor pens measuring 2.0 × 2.0 m, with concrete floors covered with wood shavings litter. Supplemental heating was provided by 250-W infrared lamps during the first 21 d of age. On the first day of life, all birds were vaccinated against coccidiosis by spray application of 20 mL vaccine solution per pen, according to the manufacturer’s recommendations.

Birds remained housed indoors until 28 d of age. From 29 d onwards, birds had access to outdoor paddocks measuring 30 m² per pen, enclosed with galvanised wire mesh and covered with Tifton grass (*Cynodon* spp.), characterising a semi-intensive free-range system. Birds had access to the outdoor area during the daytime and were housed indoors during the night. Drinkers were cleaned daily, and feed was replenished as required. Growth performance was evaluated at 70, 77, and 84 d of age by measuring body weight (BW), feed intake (FI), and feed conversion ratio (FCR) as indicated by Valentim et al. (2018). Body weight was determined by weighing all birds within each experimental unit at each evaluation age. Feed intake was calculated as the difference between the amount of feed offered and feed refusals recorded in each pen during the corresponding experimental period. Feed conversion ratio was calculated as the ratio between feed intake and body weight gain for each experimental unit. Feed intake and feed conversion ratio were corrected for mortality when necessary. For carcass and meat quality evaluations, birds were slaughtered at 70, 77, and 84 d of age. At each slaughter age, four birds per experimental unit with body weights close to the pen average were selected and individually identified using leg bands, considering a variation of ± 5% from the average body weight at 70 and 77 d, and ± 10% at 84 d. Birds were subjected to feed withdrawal for 10 h and water withdrawal for 30 min before slaughter, after which fasting live body weight was recorded. Birds were slaughtered by cervical dislocation followed by exsanguination through carotid artery severance. Carcasses were scalded in chlorinated water at 60 °C and defeathered using a mechanical plucker. Carcass traits evaluated included fasting live body weight (g), carcass yield (%), breast yield (%), and thigh–drumstick yield (%). Carcass yield was calculated as the percentage of eviscerated carcass weight without head and feet relative to fasting live body weight. Breast and thigh–drumstick yields were expressed relative to eviscerated carcass weight (Valentim et al. 2017). For meat quality evaluation, two breast muscle samples per experimental unit were randomly collected on the day of slaughter and stored at 4 °C for 24 h prior to analysis. The evaluated parameters included lightness (L*), redness (a*), yellowness (b*), chroma (C), hue angle (h°), pH, cooking loss, water-holding capacity, and shear force as indicated by de Castilho Heiss et al. (2025). Muscle pH was measured using a digital pH meter (mPA-210, MS Tecnopon, Piracicaba, SP, Brazil) equipped with a penetration electrode, with analyses performed in triplicate. Meat colour (L*, a*, and b*) was measured on the ventral surface of the breast muscle using a CR-400 Chroma Meter (Konica Minolta, Tokyo, Japan), with measurements also performed in triplicate. Chroma and hue angle were calculated according to the following equations:

$${\mathrm{C}} = \sqrt{a^2 + b^2}$$

$$h^\circ = \arctan\left({b}/{a}\right)^{\star\star}$$

Water-holding capacity was determined using a 0.5-g breast muscle sample placed between filter papers and glass plates (12 × 12 × 1 cm) under a 10-kg load for 5 min, with values calculated based on sample weight loss during compression.

Cooking loss was determined using approximately 50 g of breast muscle wrapped in aluminium foil and heated until reaching an internal temperature between 82 and 85 °C at the geometric centre of the sample. After cooling to room temperature, samples were reweighed, and cooking loss was calculated as the difference between initial and final sample weight.

Shear force was measured using a TA-XT Plus Texture Analyser (Stable Micro Systems, Surrey, UK). Samples measuring 1 × 1 × 2 cm obtained after cooking loss determination were used. Maximum positive peak values were obtained using Exponent Lite version 5.1 software, and results were expressed as kgf cm⁻².

### Statistical analysis

Data were analysed according to a completely randomised 4 × 2 factorial design, considering the fixed effects of strain, sex, and their interaction. Growth performance variables (BW, FI, and FCR) were analysed separately at each evaluation age (70, 77, and 84 d). Carcass traits and meat quality parameters were also analysed separately for each slaughter age.

The experimental unit for growth performance analysis was the pen, consisting of 27 birds. For carcass and meat quality analyses, the experimental unit was defined as the mean value obtained from the birds sampled within each pen.

Prior to statistical analysis, assumptions of normality of residuals, homogeneity of variances, and independence of errors were verified. When significant effects were detected, means were compared using Tukey’s multiple comparison test at a significance level of *P* < 0.05. Statistical analyses were performed using the MIXED procedure of the Statistical Analysis System (SAS Institute Inc., Cary, NC, USA; 2021).

## Results

Environmental temperatures recorded during the experimental period ranged from 14.2 to 25.3 °C at 70 d, 14.4 to 26.2 °C at 77 d, and 14.1 to 25.5 °C at 84 d of age. No significant interaction between sex and strain was observed for growth performance variables at any evaluated age (*P* > 0.05). However, significant effects of sex and strain were detected for body weight and feed intake at 70, 77, and 84 d of age (*P* < 0.01; Table 2), with males and the Hubbard strains (Pesadão Hubbard and Pescoço Pelado Hubbard) consistently showing higher values.

**Table 2:** Growth performance of slow-growing broilers at 70, 77, and 84 d of age according to sex and strain

Variable	Sex		Strain				*p* value			
	Male	Female	PPH	PPS	PH	PS	S	L	S×L	
70 days of age										
BW	3150.56 ± 58.00^a^	2534.81 ± 63.40^b^	2957.63 ± 353.99 A	2591.50 ± 299.95 C	3039.12 ± 335.82 A	2782.50 ± 349.73B	< 0.0001	< 0.0001	0.355	
FI	8169.88 ± 223.79^a^	7079.19 ± 296.08^b^	7829.62 ± 606.47 A	7094.87 ± 605.63B	8148.37 ± 625.97 A	7425.25 ± 697.79B	< 0.0001	< 0.0001	0.970	
FCR	2.62 ± 0.06^b^	2.83 ± 0.06^a^	2.69 ± 0.14	2.78 ± 0.10	2.72 ± 0.13	2.71 ± 0.14	< 0.0001	0.104	0.781	
77 days of age										
BW	3484.88 ± 70.09^a^	2771.69 ± 74.32^b^	3263.50 ± 430.71 A	2843.75 ± 358.19 C	3322.25 ± 410.74 A	3083.62 ± 354.09B	< 0.0001	< 0.0001	0.163	
FI	9712.7 ± 270.73^a^	8365.3 ± 372.83^b^	9249.12 ± 792.36AB	8421.50 ± 768.67 C	9693.75 ± 746.22 A	8791.50 ± 812.16BC	< 0.0001	< 0.0001	0.978	
FCR	2.82 ± 0.08^b^	3.05 ± 0.06^a^	2.88 ± 0.17B	3.01 ± 0.13 A	2.96 ± 0.18AB	2.89 ± 0.11AB	< 0.0001	0.0206	0.349	
84 days of age										
BW	3787.13 ± 104.73^a^	2995.69 ± 115.88^b^	3536.12 ± 469.31 A	3117.25 ± 427.93B	3621.12 ± 460.05 A	3291.12 ± 398.92B	< 0.0001	< 0.0001	0.788	
FI	11256.7 ± 459.91^a^	9622.9 ± 425.72^b^	10738.00 ± 914.16AB	9760.75 ± 984.23 C	11155.75 ± 985.70 A	10104.62 ± 1011.86BC	< 0.0001	< 0.0001	0.948	
FCR	3.00 ± 0.07^b^	3.25 ± 0.08^a^	3.08 ± 0.18	3.18 ± 0.15	3.12 ± 0.19	3.11 ± 0.11	< 0.0001	0.1871	0.363	

PPH = Pescoço Pelado Hubbard; PPS = Pescoço Pelado Sasso; PH = Pesadão Hubbard; PS = Pesadão Sasso; BW = body weight; FI = feed intake; FCR = feed conversion ratio; S = sex; L = strain; S × L = interaction between sex and strain. Values are means ± SD. Means within the same row followed by different lowercase letters (sex effect) or uppercase letters (strain effect) differ significantly according to Tukey’s test (*P* < 0.05)

At 70 d of age, feed conversion ratio was affected only by sex (*P* < 0.01), with males showing better feed efficiency than females. At 77 d, feed conversion ratio was influenced by both sex and strain (*P* < 0.05), with males presenting lower feed conversion ratios than females. Among strains, Pescoço Pelado Hubbard showed better feed conversion than Pescoço Pelado Sasso, without differing from Pesadão Hubbard and Pesadão Sasso. At 84 d, feed conversion ratio was affected only by sex (*P* < 0.01), with males again showing superior feed efficiency.

Overall, males consistently presented greater body weight, higher feed intake, and better feed conversion than females across all evaluated ages. Sex and strain significantly influenced carcass characteristics across slaughter ages (Table 3). Fasting body weight was affected by both factors at all evaluated ages (*P* < 0.01), with males and the Hubbard strains showing higher values.

**Table 3:** Carcass characteristics of slow-growing broilers at 70, 77, and 84 d of age according to sex and strain

Variable	Sex		Strain				*p* value		
	Male	Female	PPH	PPS	PH	PS	S	L	S×L
70 days of age									
BW	3154.38 ± 93.78^a^	2542.50 ± 88.60^b^	2977.50 ± 369.23 A	2585.00 ± 320.40 C	3015.00 ± 308.91 A	2816.25 ± 364.49B	< 0.0001	< 0.0001	0.731
CY	70.34 ± 0.98	70.13 ± 1.07	71.94 ± 0.82 A	68.82 ± 0.74 C	70.57 ± 1.30AB	69.61 ± 1.46BC	0.5934	< 0.0001	0.335
BY	31.91 ± 0.66^b^	33.67 ± 1.69^a^	34.23 ± 1.40 A	31.06 ± 1.08B	33.69 ± 1.69 A	32.18 ± 2.68AB	0.0052	0.0025	0.586
TDY	31.51 ± 0.90^a^	30.18 ± 0.75^b^	30.65 ± 0.67B	31.90 ± 0.73 A	30.42 ± 1.67B	30.40 ± 1.32B	0.0064	0.0003	0.072
77 days of age									
BW	3397.5 ± 128.54^a^	2705.00 ± 89.98^b^	3162.50 ± 437.90AB	2745.00 ± 350.50 C	3282.50 ± 365.26 A	3015.00 ± 386.96B	< 0.0001	< 0.0001	0.5148
CY	69.50 ± 0.76	68.98 ± 0.73	70.94 ± 1.23	67.94 ± 0.73	69.47 ± 0.91	68.59 ± 0.74	0.0776	< 0.0001	0.0313
BY	31.95 ± 0.73^b^	34.24 ± 0.65^a^	34.08 ± 1.16 A	30.97 ± 1.20B	33.99 ± 1.69 A	33.35 ± 1.54 A	< 0.0001	< 0.0001	0.4002
TDY	32.82 ± 1.48^a^	30.90± 0.51^b^	32.24 ± 1.31	32.74 ± 1.02	31.57 ± 1.65	30.91 ± 2.53	0.0011	0.0947	0.7402
84 days of age									
BW	3698.75 ± 135.84^a^	2892.50 ± 154.60^b^	3467.50 ± 521.63 A	3026.25 ± 368.45B	3516.25 ± 425.81 A	3172.50 ± 478.77B	< 0.0001	< 0.0001	0.5046
CY	70.78 ± 1.28^a^	69.61 ± 1.41^b^	71.59 ± 2.42 A	69.34 ± 1.01B	69.93 ± 1.13AB	69.91 ± 1.84AB	0.0467	0.0468	0.2218
BY	30.95 ± 0.78^b^	34.27 ± 0.84^a^	34.13 ± 1.68 A	30.61 ± 2.03 C	33.40 ± 2.53AB	32.29 ± 1.58B	< 0.0001	< 0.0001	0.1065
TDY	33.97 ± 0.87^a^	31.47 ± 1.34^b^	32.55 ± 1.66	33.23 ± 1.63	32.77 ± 2.06	32.32 ± 1.59	< 0.0001	0.4854	0.8627

PPH = Pescoço Pelado Hubbard; PPS = Pescoço Pelado Sasso; PH = Pesadão Hubbard; PS = Pesadão Sasso; BW = fasting body weight; CY = carcass yield; BY = breast yield; TDY = thigh–drumstick yield; S = sex; L = strain; S × L = interaction between sex and strain. Values are means ± SD. Means within the same row followed by different lowercase letters (sex effect) or uppercase letters (strain effect) differ significantly according to Tukey’s test (*P* < 0.05)

At 70 d, carcass yield was influenced by strain (*P* < 0.01), with Pescoço Pelado Hubbard showing higher yield than Pescoço Pelado Sasso and Pesadão Sasso, without differing from Pesadão Hubbard. Breast yield was affected by sex and strain (*P* < 0.05), with females presenting higher values than males, and higher breast yield observed in Pescoço Pelado Hubbard and Pesadão Hubbard. Thigh–drumstick yield was also influenced by sex and strain (*P* < 0.05), with males showing higher values and Pescoço Pelado Sasso presenting the highest yield among strains.

At 77 d, a significant interaction between sex and strain was observed for carcass yield (*P* < 0.05; Table 4). Within the Pescoço Pelado Hubbard strain, males showed greater carcass yield than females, whereas no differences between sexes were observed within the Pescoço Pelado Sasso, Pesadão Hubbard, or Pesadão Sasso strains. Within each sex, Pescoço Pelado Hubbard showed the highest carcass yield, whereas Pescoço Pelado Sasso showed the lowest values. Breast yield was influenced by sex and strain (*P* < 0.01), with females showing higher values and Pescoço Pelado Sasso presenting the lowest breast yield. Thigh–drumstick yield was affected only by sex (*P* < 0.01), with males presenting higher values.

**Table 4:** Interaction between sex and strain for carcass yield of slow-growing broilers at 77 d of age

Sex	Strain			
	PPH	PPS	PH	PS
Male	71.99 ± 0.24 aA	67.94 ± 0.96 aC	69.44 ± 1.16 aB	68.63 ± 0.71 aBC
Female	69.88 ± 0.73 bA	67.95 ± 0.57 aC	69.51 ± 0.77 aB	68.50 ± 0.88 aBC

PPH = Pescoço Pelado Hubbard; PPS = Pescoço Pelado Sasso; PH = Pesadão Hubbard; PS = Pesadão Sasso. Values are means ± SD. Different lowercase letters within columns and uppercase letters within rows indicate significant differences according to Tukey’s test (*P* < 0.05)

At 84 d, carcass yield was influenced by both sex and strain (*P* < 0.05), with males showing higher values than females. Among strains, Pescoço Pelado Hubbard showed higher carcass yield than Pescoço Pelado Sasso, without differing from Pesadão Hubbard and Pesadão Sasso. Breast yield was affected by sex and strain (*P* < 0.01), with females showing higher values, and greater breast yield observed in Pescoço Pelado Hubbard and Pesadão Hubbard. Thigh–drumstick yield was affected only by sex (*P* < 0.01), with males presenting higher values.

At 70 d (Table 5), sex affected b* and h° values (*P* < 0.01), with males showing higher values than females. Muscle pH was influenced by both sex and strain (*P* < 0.05), with higher values observed in males and in the Pesadão Sasso strain. No significant effects were detected for L*, a*, chroma, cooking loss, water-holding capacity, or shear force.

**Table 5:** Breast meat quality parameters of slow-growing broilers at 70 and 77 d of age according to sex and strain

Variable	Sex		Strain				*p* value		
	Male	Female	PPH	PPS	PH	PS	S	L	S×L
70 days of age									
L*	47.56 ± 1.46	48.22 ± 1.33	48.49 ± 1.76	48.38 ± 1.00	48.05 ± 1.78	46.63 ± 1.18	0.2236	0.0716	0.6027
a*	4.41 ± 0.75	4.36 ± 0.57	4.06 ± 1.06	4.46 ± 0.60	4.33 ± 0.78	4.70 ± 0.30	0.8563	0.4047	0.3463
b*	2.42 ± 0.70^a^	1.25 ± 0.51^b^	2.08 ± 1.28	1.42 ± 0.59	1.90 ± 0.66	1.94 ± 0.98	< 0.0001	0.2461	0.1995
C	5.15 ± 0.78	4.60 ± 0.57	4.68 ± 1.33	4.76 ± 0.50	4.81 ± 0.86	5.25 ± 0.43	0.0655	0.5026	0.2106
h*	28.22 ± 8.47^a^	16.49 ± 6.37^b^	25.52 ± 12.13	18.75 ± 9.11	23.54 ± 8.17	21.60 ± 9.59	0.0002	0.3612	0.3748
pH	5.82 ± 0.05^a^	5.75 ± 0.03^b^	5.75 ± 0.04B	5.80 ± 0.07AB	5.76 ± 0.06AB	5.83 ± 0.07 A	0.0054	0.0334	0.5724
CL	0.45 ± 0.02	0.45 ± 0.01	0.45 ± 0.02	0.45 ± 0.02	0.45 ± 0.02	0.46 ± 0.02	0.9685	0.6747	0.6846
WHC	0.28 ± 0.01	0.28 ± 0.02	0.28 ± 0.02	0.26 ± 0.03	0.28 ± 0.02	0.29 ± 0.01	0.6473	0.1468	0.3119
SF	0.75 ± 0.09	0.74 ± 0.18	0.81 ± 0.14	0.67 ± 0.14	0.73 ± 0.13	0.77 ± 0.15	0.9770	0.2964	0.7606
77 days of age									
L*	48.42 ± 1.34^b^	49.98 ± 1.12^a^	48.90 ± 1.84	49.14 ± 1.37	50.11 ± 1.39	48.65 ± 1.27	0.0019	0.1339	0.4781
a*	4.14 ± 0.53^a^	3.64 ± 0.45^b^	3.85 ± 0.78	4.08 ± 0.69	3.61 ± 0.54	4.03 ± 0.33	0.0132	0.3018	0.1409
b*	2.49 ± 0.77^a^	1.32 ± 0.57^b^	2.11 ± 1.15 A	1.02 ± 0.69B	1.77 ± 0.90AB	2.72 ± 0.91 A	0.0001	0.0011	0.8773
C	4.97 ± 0.71^a^	4.02 ± 0.46^b^	4.55 ± 1.07	4.32 ± 0.73	4.14 ± 0.81	4.96 ± 0.61	0.0004	0.0918	0.2444
h*	29.96 ± 7.13^a^	19.60 ± 7.86^b^	27.77 ± 12.26 A	13.99 ± 8.92B	24.35 ± 8.84AB	33.03 ± 8.34 A	0.0017	0.0010	0.5564
pH	5.90 ± 0.06^a^	5.85 ± 0.06^b^	5.94 ± 0.07 A	5.83 ± 0.05B	5.82 ± 0.06B	5.89 ± 0.08AB	0.0417	0.0066	0.9693
CL	0.45 ± 0.02	0.44 ± 0.01	0.42 ± 0.02B	0.46 ± 0.01 A	0.45 ± 0.02AB	0.45 ± 0.02AB	0.6071	0.0224	0.6288
WHC	0.30 ± 0.01	0.29 ± 0.02	0.30 ± 0.02	0.28 ± 0.02	0.29 ± 0.03	0.30 ± 0.03	0.3544	0.4075	0.4105
SF	1.95 ± 0.39	1.85 ± 0.36	1.79 ± 0.45AB	2.31 ± 0.39 A	1.57 ± 0.35B	1.94 ± 0.38AB	0.4848	0.0094	0.4841

PPH = Pescoço Pelado Hubbard; PPS = Pescoço Pelado Sasso; PH = Pesadão Hubbard; PS = Pesadão Sasso; L* = lightness; a = redness; b* = yellowness; C = chroma; h° = hue angle; CL = cooking loss; WHC = water-holding capacity; SF = shear force; S = sex; L = strain; S × L = interaction between sex and strain

At 77 d (Table 5), L*, a*, and chroma were affected by sex (*P* < 0.05), with females showing higher L* values and males presenting higher a* and chroma values. Variables b*, h°, and pH were influenced by both sex and strain (*P* < 0.05). Cooking loss and shear force were affected by strain (*P* < 0.05), with Pescoço Pelado Hubbard showing lower cooking loss than Pescoço Pelado Sasso, while the other strains showed intermediate values, and higher shear force observed in Pescoço Pelado Sasso.

At 84 d (Table 6), L* and b* values were affected by strain (*P* < 0.05). Hue angle (h°) was influenced by both sex and strain (*P* < 0.01), with males showing higher values. A significant interaction between sex and strain was observed for cooking loss (*P* < 0.05; Table 7). Within the Pesadão Sasso strain, females showed greater cooking loss than males, whereas no differences between sexes were observed within the other strains. Among females, Pesadão Sasso birds showed greater cooking loss than females of the Pescoço Pelado Hubbard and Pescoço Pelado Sasso strains, while Pesadão Hubbard females showed intermediate values. No differences among strains were observed within males.

**Table 6:** Breast meat quality parameters of slow-growing broilers at 84 d of age according to sex and strain

Variables	Sex		Strain				*p* value		
	Male	Female	PPH	PPS	PH	PS	S	L	S×L
L*	49.15 ± 1.37	49.41 ± 1.28	50.52 ± 1.22 A	49.28 ± 1.98AB	49.20 ± 1.09AB	48.13 ± 0.94B	0.6145	0.0263	0.8865
a*	3.80 ± 0.70	3.79 ± 0.60	3.28 ± 0.54	3.91 ± 0.72	3.95 ± 0.90	4.04 ± 0.37	0.9537	0.1407	0.6306
b*	1.87 ± 0.41	1.29 ± 0.82	1.22 ± 0.69B	0.89 ± 0.39B	2.40 ± 1.31 A	1.81 ± 0.92AB	0.0604	0.0068	0.2194
C	4.36 ± 0.70	4.16 ± 0.87	3.63 ± 0.50	4.09 ± 0.70	4.79 ± 1.50	4.50 ± 0.54	0.5599	0.1067	0.8918
h*	26.33 ± 5.56^a^	17.01 ± 6.92^b^	19.59 ± 10.74BC	14.11 ± 6.91 C	28.94 ± 5.06 A	24.03 ± 10.82AB	0.0006	0.0013	0.0610
pH	5.82 ± 0.05	5.78 ± 0.04	5.80 ± 0.07	5.78 ± 0.04	5.77 ± 0.08	5.85 ± 0.06	0.1027	0.1129	0.9464
CL	0.23 ± 0.01	0.23 ± 0.02	0.241 ± 0.03	0.22 ± 0.03	0.23 ± 0.03	0.23 ± 0.03	0.3674	0.3731	0.0151
WHC	0.31 ± 0.02	0.33 ± 0.03	0.33 ± 0.02	0.31 ± 0.02	0.33 ± 0.02	0.32 ± 0.05	0.1931	0.6917	0.9470
SF	2.39 ± 0.98	2.04 ± 0.67	2.30± 0.70	2.41 ± 1.03	2.12 ± 1.02	2.02 ± 0.64	0.2703	0.8111	0.5842

PPH = Pescoço Pelado Hubbard; PPS = Pescoço Pelado Sasso; PH = Pesadão Hubbard; PS = Pesadão Sasso; L* = lightness; a = redness; b* = yellowness; C = chroma; h° = hue angle; CL = cooking loss; WHC = water-holding capacity; SF = shear force; S = sex; L = strain; S × L = interaction between sex and strain

**Table 7:** Interaction between sex and strain for cooking loss of slow-growing broilers at 84 d of age

Sex	Strain			
	PPH	PPS	PH	PS
Male	0.25 ± 0.03 bB	0.23 ± 0.008 bB	0.21 ± 0.03 bB	0.21 ± 0.01 bB
Female	0.22 ± 0.02 bB	0.21 ± 0.04 bB	0.24 ± 0.02 bAB	0.26 ± 0.01 aA

Values are means ± SD. Different lowercase letters within columns and uppercase letters within rows indicate significant differences according to Tukey’s test (*P* < 0.05)

## Discussion

The superior growth performance observed in males, characterised by greater body weight, higher feed intake, and improved feed conversion ratio, may be attributed to physiological and hormonal differences between sexes. Api et al. (2017) reported that males generally exhibit greater efficiency in nutrient utilisation, which may be associated with differences in digestive capacity and metabolic activity. These factors favour increased muscle deposition and faster growth compared with females.

Hormonal regulation also plays an important role in growth performance. Purchas et al. (2002) described that testosterone stimulates protein synthesis and muscle development, whereas females tend to deposit proportionally more adipose tissue due to estrogen influence, particularly with the onset of sexual maturity. This difference in tissue accretion likely explains the greater body weight and superior feed efficiency observed in males in the present study.

The present findings are consistent with those reported by Del Castilho et al. (2013), who also observed greater body weight and improved feed conversion in male slow-growing broilers at comparable slaughter ages. Similar genotype-related differences in growth performance have also been reported by Faria et al. (2009). Similarly, Veloso et al. (2015) reported that strains with greater feed intake tend to achieve higher final body weight, which agrees with the superior productive performance observed in the Hubbard strains (Pesadão Hubbard and Pescoço Pelado Hubbard) in the present study. These differences reinforce the importance of genotype selection when production efficiency is a primary objective in alternative poultry systems.

Sex-related differences in carcass composition observed in this study are also consistent with known physiological differences in tissue deposition. The greater carcass yield and thigh–drumstick yield observed in males, particularly at later slaughter ages, suggest greater lean tissue accretion, as proposed by Api et al. (2017). Mahgoub et al. (2004) reported that hormonal differences strongly influence carcass composition, with males tending to exhibit greater muscle development, whereas females often present relatively greater fat deposition and breast development. This pattern was confirmed in the present study, where females consistently showed higher breast yield, while males presented greater thigh–drumstick yield. Similar results were reported by Santos et al. (2005), who observed greater leg cut yields in male birds from alternative broiler strains.

Differences among strains in carcass characteristics further confirm the direct influence of genotype on productive performance. Similar genotype-dependent differences in carcass yield and cut distribution were reported by Rizzi et al. (2009). Veloso et al. (2015) demonstrated that slow-growing strains may exhibit distinct growth patterns and carcass composition, which directly affects their suitability for specific production systems and market preferences. In the present study, the superior carcass performance of Hubbard strains suggests greater biological efficiency under the evaluated free-range conditions.

Importantly, the significant interaction between sex and strain for carcass yield at 77 d demonstrates that the effect of sex was genotype-dependent rather than uniform across all strains. Males showed greater carcass yield than females only within the Pescoço Pelado Hubbard strain, whereas no sex-related differences were detected within the other genotypes. This finding suggests that the magnitude of sexual dimorphism in carcass development may depend on genetic background and become evident at specific stages of growth. The superior carcass yield of Pescoço Pelado Hubbard males indicates a genotype-specific advantage that would have been obscured if only the overall main effects of sex and strain had been considered. From a practical perspective, this interaction suggests that sex-specific slaughter strategies may be advantageous for some genotypes but unnecessary for others. Thus, producers targeting carcass yield may benefit from considering both genotype and sex when defining slaughter schedules, particularly around 77 d of age.

Meat quality traits were moderately influenced by sex and strain, particularly colour parameters and selected texture-related traits. The greater L* values observed in females at 77 d may be associated with differences in postmortem muscle metabolism and pH decline. According to Gaya et al. (2006), lower muscle pH may increase light scattering at the meat surface, resulting in paler meat. Ramos and Gomide (2017) further emphasised that postmortem pH decline directly affects water retention and colour development in meat. Vorel et al. (2026) also reported that muscle pH is associated with glycogen reserves at slaughter, which directly influence colour development and water retention capacity.

Sex also influenced colour coordinates, with males showing higher b* and h° values at specific slaughter ages. These differences may reflect variation in muscle fibre characteristics, pigment concentration, and fat deposition between sexes, as discussed by Bonamigo and Molento (2012). Veloso et al. (2015) similarly reported that both genotype and sex may influence colour attributes in alternative broiler strains.

Texture-related traits were influenced primarily by genotype at specific ages, suggesting differences in muscle structural characteristics among strains. Monahan et al. (2005) also highlighted that postmortem biochemical changes can influence moisture retention and texture development in poultry meat. Hughes et al. (2014) described that water retention properties are closely associated with meat tenderness and juiciness, while lower water retention may contribute to firmer texture. Similar relationships between moisture retention and meat texture were reported by Jonsäll et al. (2001). In the present study, higher shear force values in Pescoço Pelado Sasso at 77 d suggest reduced tenderness compared with other strains.

The significant interaction between sex and strain for cooking loss at 84 d further demonstrates that meat quality responses cannot be interpreted solely from the independent effects of sex or genotype. Females of the Pesadão Sasso strain showed greater cooking loss than males of the same strain, whereas no sex-related differences were detected within the other genotypes. Moreover, among females, Pesadão Sasso birds exhibited greater cooking loss than both Pescoço Pelado strains. This genotype-specific response suggests differences in the ability of muscle tissue to retain moisture during heating, potentially reflecting variation in muscle composition, fibre characteristics, or postmortem biochemical processes. From a technological perspective, greater cooking loss may reduce cooked product yield and negatively affect juiciness, thereby influencing both processing efficiency and consumer acceptance. Therefore, the interaction observed at 84 d has practical implications for genotype- and sex-specific slaughter decisions when meat processing yield and moisture retention are production priorities.

Taken together, the present results demonstrate that both sex and genotype are important determinants of productive performance, carcass composition, and selected meat quality traits in slow-growing broilers reared under free-range conditions. More importantly, the significant interactions observed for carcass yield at 77 d and cooking loss at 84 d demonstrate that sex-related responses are not consistent across genotypes at the evaluated ages. The greater carcass yield of Pescoço Pelado Hubbard males at 77 d indicates a genotype-specific productive advantage, whereas the greater cooking loss of Pesadão Sasso females at 84 d reveals a sex-specific limitation in meat processing yield. These findings highlight the importance of considering interactions between biological factors rather than relying exclusively on their main effects when selecting genotypes and defining slaughter strategies for alternative poultry production systems.

## Conclusion

Sex and genotype significantly influenced growth performance, carcass traits, and selected breast meat quality parameters of slow-growing broilers reared in a free-range system. Males showed superior growth performance and greater thigh–drumstick yield, whereas females consistently presented higher breast yield. Among the evaluated strains, Pescoço Pelado Hubbard and Pesadão Hubbard demonstrated greater productive efficiency under the experimental conditions. However, the effects of sex were genotype-dependent for specific economically relevant traits. At 77 d, males had greater carcass yield than females only within the Pescoço Pelado Hubbard strain, whereas at 84 d, Pesadão Sasso females showed greater cooking loss than males of the same strain. These interactions demonstrate that genotype, sex, and slaughter age should be considered jointly when defining production and slaughter strategies. Pescoço Pelado Hubbard males may be advantageous when carcass yield is prioritised at 77 d, whereas slaughtering Pesadão Sasso females at 84 d may be less desirable when minimising cooking loss and maximising processing yield are primary objectives.

## Data Availability

The datasets generated during and/or analyzed during the current study are not publicly available but are available from the corresponding author upon reasonable request.

## Declarations

### Animal welfare

This study was conducted in accordance with the ethical principles for animal experimentation and welfare. All experimental procedures were reviewed and approved by the Animal Use Ethics Committee of the Federal University of Jequitinhonha and Mucuri Valleys (UFVJM), under protocol number 12/2020.
